# Heart rate variability, daily cortisol indices and their association with psychometric characteristics and gut microbiota composition in an Italian community sample

**DOI:** 10.1038/s41598-025-93137-8

**Published:** 2025-03-12

**Authors:** Sebastiano Ravenda, Leonardo Mancabelli, Sara Gambetta, Margherita Barbetti, Francesca Turroni, Luca Carnevali, Marco Ventura, Andrea Sgoifo

**Affiliations:** 1 Department of Chemistry, Life Sciences and Environmental Sustainability, Stress Physiology Lab, University of Parma, Parma, Italy; 2 Department of Medicine and Surgery, University of Parma, Parma, Italy; 3 Microbiome Research Hub, University of Parma, Parma, Italy; 4 Department of Chemistry, Life Sciences, and Environmental Sustainability, Laboratory of Probiogenomics, University of Parma, Parma, Italy

**Keywords:** HRV, Cortisol, Gut, Microbiota, Stress, Depression, Microbiology, Physiology, Psychology

## Abstract

The microbiota–gut–brain axis is a complex communication system that plays a crucial role in influencing various aspects of our physical and mental health. The goal of this study was to determine the extent to which individual differences in resting measures of vagally-mediated heart rate variability (HRV) and cortisol levels were associated with psychometric and specific gut microbiota characteristics in seventy-five (38 females) healthy individuals. Participants were assessed for vagally-mediated HRV, daily salivary cortisol levels, psychometric characteristics, and gut microbiota composition. Using a categorical approach based on the median split of HRV and cortisol values, we identified an association between low vagally-mediated HRV, greater depressive symptomatology, and altered gut microbiota (e.g., a higher abundance of *Prevotella* and a smaller abundance of *Faecalibacterium*,* Alistipes*, and *Gemmiger).* This suggests that vagally-mediated HRV may be a useful biomarker of microbiota-gut brain axis function, and that low vagally-mediated HRV may play an important role in the bidirectional link between gut dysbiosis and depression. On the other hand, daily cortisol parameters (e.g., cortisol awakening response, diurnal cortisol slope) were associated either with higher anxiety and perceived stress, or with a specific gut microbiota profile. Therefore, their utility as biomarkers of microbiota-gut-brain axis function needs further scrutiny.

**Supplementary Information:**

The online version contains supplementary material available at 10.1038/s41598-025-93137-8.

## Introduction

Trillions of gut bacteria, collectively known as gut microbiota, and the brain are in bidirectional communication and can reciprocally influence each other’s activity^1^. As such, the microbiota is a crucial component of the gut-brain axis. Disruptions in this axis affect intestinal motility and secretion, leading, for example, to the development of inflammatory bowel disease (IBD), but are also associated with altered responses to acute and chronic stress, behavioral changes, and vulnerability to psychological disorders^1^. Studies have associated gastrointestinal diseases (e.g., IBD) with psychological comorbidities, and have shown that several stress-related psychiatric disorders, like depression and anxiety, have well-established links to intestinal dysbiosis^2^. Furthermore, preclinical models have demonstrated that gut microbiota disturbances occur in rodents exhibiting anxiety- and depressive- like behaviors (e.g^3^). , and that both behavioral and microbial alterations are normalized after administration of bacterial probiotics^4^.

The vagus nerve is a key mediator of the microbiota–gut–brain axis, allowing the brain to influence intestinal activities and microbiota composition, and the gut to influence mood, cognition, and mental health^5^. Moreover, higher cardiac vagal activity is associated with health and well-being, whereas lower cardiac vagal activity relates to morbidity, mortality, and stress related disorders^5–8^. Using heart rate variability (HRV) as a surrogate measure of cardiac vagal function, studies have found that low vagally-mediated HRV is a shared endophenotype of both psychiatric and gastrointestinal disorders^8,9^. Also, preliminary studies have demonstrated an association between HRV, gut microbiota profiles, and psychological characteristics in non-clinical populations. For example, individuals with low vagally-mediated HRV were found to have a worse psychological profile associated with a reduced alpha diversity and a higher abundance of bacteria related to intestinal pathologies such as IBD^5,10–12^. This suggests that HRV may represent a promising biomarker for microbiota-gut-brain axis imbalance.

The hypothalamic-pituitary-adrenal axis, with its primary end-product cortisol, is another key mediator of brain-gut communication^1^. Individuals with irritable bowel syndrome (IBS) have been shown to have altered serum cortisol levels associated with changes in gut microbiota composition^13^. Other preliminary studies have found that hair and urine cortisol levels correlate with different microbiota profiles and psychological outcomes^10,14^. Also, salivary cortisol levels show associations with different gut microbiota profiles^15–17^. For example, young students with a microbiota dominated by *Lactobacillus* and *Bifidobacterium* exhibited lower levels of salivary cortisol compared with students with a microbiota dominated by *Bacteroides*^17^. Therefore, cortisol levels may represent important determinants of optimal microbiota-gut-brain axis activity.

However, the few human studies that investigated the association between individual differences in vagally-mediated HRV, cortisol levels, psychological characteristics, and microbiota profile have several limitations. For instance, some focused exclusively on one sex (e.g., females^11^) specific age-groups (e.g., children^10,15,16^), or clinical cohorts (e.g., patients with depression^12^), while others did not consider confounding factors such as body mass index^11^. Additionally, to the best of our knowledge, HRV and cortisol levels have never been analyzed in combination with psychological characteristics and gut microbiota within a sample of healthy individuals. Therefore, the main goal of this study was to determine the extent to which individual differences in vagally-mediated HRV and daily measures of salivary cortisol are associated with psychometric characteristics and specific gut microbiota features in a group of healthy individuals recruited within the urban area of Parma (Italy).

## Method

### Participants

The present study is based on a secondary analysis of data obtained from Italian healthy adults recruited for the Parma Microbiota project^18^. The project was approved by the local ethics Committee (Comitato Etico dell’Area Vasta Emilia Nord, Emilia-Romagna Region, Italy, under the ID 1107/2020/TESS/UNIPR) and all procedures were performed in accordance with the Declaration of Helsinki. All participants provided written informed consent. For the aim of this study, we considered a subsample of participants (*n* = 80) of the project from whom salivary samples and HRV data were collected. Inclusion criteria included being resident in the province of Parma (Italy), being older than 18, and absence of current or past psychiatric or cardiac disorders, IBD, or other inflammatory pathologies. Exclusion criteria included the presence, at the time of the laboratory assessment, of gastrointestinal symptoms/signs (diarrhea, abdominal pain, constipation, etc.) or systemic symptoms (low-grade fever, arthralgia) suggestive of the presence of IBD or other unknown acute or chronic gastroenterological pathologies, and use of antibiotic therapies during the 20 days prior to the laboratory assessment. After exclusion of five participants because of missing cortisol data, the final sample consisted of 75 healthy subjects (age (SD): 36 (14) years; 38 females).

### Procedure

Participants were asked to refrain from physical activity, caffeine consumption or smoking at least 2 h prior to their arrival to the lab (between 2 pm and 5 pm). After signing the consent form, they completed a series of socio-demographic, lifestyle, and dispositional scales (see below “Psychometric questionnaires”). Subsequently, they were fitted with the Firstbeat Bodyguard 2 device (Firstbeat Technologies, Finland) for recordings of R-R intervals. Recordings lasted for 10 min while the participants sat quietly on a comfortable chair. Subsequently, participants were given stool collection tubes, saliva cotton swabs, and saliva collection tubes (Salimetrics, Cambridge, UK). Participants were instructed orally and were also given written instructions on how to collect and store one stool sample and four saliva samples (upon awakening, 30 min after awakening, at noon, and at 10 pm) on the next day. Importantly, they were asked to avoid eating, drinking, smoking, teeth brushing, and any physical exercise during the 30 min that preceded each saliva sample collection^19,20^. To monitor their compliance, participants were given a sheet where they had to indicate when they awoke and the exact time of each saliva sample collection. Saliva storage tubes were labeled with the exact time of collection, stored immediately after in participants’ home refrigerators, and delivered to the laboratory on the next day, alongside the handout and the sheet with the exact times of saliva sampling.

### Psychometric questionnaires

The trait version of the State-Trait Anxiety Inventory (STAI)^21^ was used to assess the severity of trait anxiety. The STAI is a 4-point Likert scale consisting of 20 items assessing how the subject feels, independent from the circumstances and status (e.g., “I feel secure,” I feel troubled”). Reliability coefficients range from 0.71 to 0.86 and internal consistency and homogeneity coefficients range between 0.83 and 0.87. State anxiety was measured using the state version of the STAI, which investigates how respondents feel “in that moment” using a 4‐point Likert scale that measures subjective feelings of apprehension, tension, nervousness, worry, and arousal. The reliability coefficient is 0.62.

The Perceived Stress Scale (PSS) was used for measuring the perception of stress over the last month^22^. Items ask how unpredictable, uncontrollable, and overloaded respondents have found their lives during the last month. Scores ranging from 14 to 26 are considered “moderate perceived stress”, those ranging from 27 to 40 are considered “high perceived stress”^22^.

The Center for Epidemiological Studies Depression Scale (CES-D) is a 20-item self-report scale designed to measure depressive symptomatology during the past week in the general population^23^. Standard cutoffs are > 16 for mild depression and > 23 for clinical depression. Cronbach’s alphas are above 0.85 in the general population and 0.90 in patients with depression confirming high reliability^23^.

### Heart rate and heart rate variability analysis

Raw R-R intervals obtained with the Firstbeat Bodyguard 2 device were analyzed with the Kubios HRV software^24^, using a medium filter for threshold-based artefact correction^25^. Separate estimates of heart rate (HR, reported in beats per minute) and HRV were generated. The root mean square of successive beat-to-beat interval differences (RMSSD, ms) was considered as a vagally-mediated index of HRV^26^. RMSSD is less susceptible to respiratory and movement artifacts compared to the alternative frequency-domain high frequency activity^27^. Throughout the remainder of this manuscript, we will use the term vagally-mediated HRV to specifically refer to RMSSD.

### Cortisol analysis

To collect saliva, participants were asked to keep oral swabs under their tongue for 2 min. Immediately after collection, saliva samples were frozen at − 20 °C. For the analysis, samples were thawed, brought to room temperature and centrifuged (1500 g × 10 min), resulting in a clear supernatant of low viscosity. Salivary cortisol levels were determined by enzyme-linked immunosorbent assay (High Sensitivity Salivary Cortisol Enzyme Immunoassay Kit: Salimetrics LLC, State College, PA). Samples were assayed in duplicates following kit instructions with a 96-well plate, using the BioTek 800 TS absorbance reader and Gen5 software (BioTek Instruments Inc., Vermont, USA). The inter-assay and intra-assay coefficients of variability were 5.9 and 8.4, respectively. Initially, mean salivary cortisol values were calculated for each collection point. Subsequently, we calculated the following parameters: the cortisol awakening response (CAR, calculated as the cortisol value 30 min after awakening minus the value upon awakening), the diurnal cortisol slope (DCS, calculated as the cortisol value upon awakening minus the value at 10 pm), and the area under the curve with respect to ground (AUC_g_, calculated as the area under the 4-time point curve).

### Gut microbiota analysis

The metagenomic data used in this study were part of datasets regarding the human gut microbiota across different life stages^28^obtained from Italian adult healthy individuals within the framework of the Parma Microbiota project. To assess the microbiota composition at the species level, the subsample of 75 healthy subjects was reanalyzed through the METAnnotatorX2 software following the standard filtering parameters reported in the manual^28,29^. In detail, the fastq files were filtered to remove reads with a quality of < 25 and to retain reads with a length of > 100 bp. Subsequently, human host DNA filtering was performed through Bowtie 2 software^30,31^, following the METAnnotatorX2 manual^32^. Afterward, the taxonomic classification of 100 000 reads was achieved by means of MegaBLAST^33^employing a manually curated and pre-processed database of genomes retrieved from the National Center for Biotechnology Information, following the METAnnotatorX2 manual^32^.

### Statistical analyses

Statistical analyses for HRV and cortisol parameters and psychometric scores were conducted using IBM SPSS Statistics for Windows version 29 (IBM Corp., Armonk, N.Y., USA). Data are expressed as means ± standard error (SE). Statistical significance was set at *p* < 0.05. The normal distribution of variables was assessed using the Kolmogorov-Smirnov test. We accounted for the non-normal distribution of psychometric scores (*p* < 0.001) by calculating their natural logarithm. For the purposes of our analyses, we used the median value of HRV (34.07 ms) to divide the population into a HIGH HRV and a LOW HRV group. Likewise, each cortisol parameter (CAR, DCS, and AUC_g_) was split at the median to form HIGH and LOW groups (CAR median value: 0.100 µg/dL; DCS median value: 0.351 µg/dL; AUC_g_ median value: 215.85 µg/dLxh). For each HRV and cortisol parameter, differences between the HIGH and LOW group were analyzed using Student’s t-tests and χ^2^tests. Mann–Whitney U tests were applied to identify differences in microbiota taxa between groups. Finally, the similarities between samples (beta-diversity) were calculated by the Bray-Curtis dissimilarity matrix based on species abundance using Emperor tool^34^. PERMANOVA analyses were performed using 999 permutations to estimate possible significant differences among groups in PCoA analyses.

## Results

### Heart rate variability, psychometric characteristics, and microbiota profile

Using the median value of HRV, participants were initially divided into LOW and HIGH HRV groups. As reported in Table 1, participants in the LOW HRV group were on average older than the HIGH HRV group (Table 1). Besides lower HRV values, the LOW HRV group had also significantly higher HR than the HIGH HRV group (Table 1). No other group differences were found in terms of general characteristics and cortisol parameters (Table 1).

**Table 1:** Participant characteristics for the LOW HRV (*n* = 37) and HIGH HRV (*n* = 38) group.

	LOW HRV	HIGH HRV	t/χ^2^	*p*	d
Self-reported gender	F = 18 M = 19	F = 20 M = 18	0.11	0.73	
Age (years)	42.3 ± 16.4	31.9 ± 10.4	3.33	< 0.01	0.07
Smokers (n)	12	10	0.26	0.6	
BMI (kg*m^−2^)	24.1 ± 4.2	23.3 ± 4.1	0.67	0.25	0.16
HR (bpm)	75.3 ± 9.4	65.8 ± 8.4	4.62	< 0.001	1.07
HRV (ms)	21.0 ± 7.5	50.3 ± 14.8	−10.83	< 0.001	− 2.52
Cortisol AW (µg/dL)	0.53 ± 0.24	0.49 ± 0.26	0.61	0.27	0.15
Cortisol AW + 30 min (µg/dL)	0.69 ± 0.39	0.64 ± 0.39	0.54	0.29	0.13
Cortisol 12:00 (µg/dL)	0.23 ± 0.16	0.26 ± 0.16	−0.67	0.25	− 0.16
Cortisol 22:00 (µg/dL)	0.11 ± 0.06	0.11 ± 0.07	−0.12	0.45	− 0.03
CAR (µg/dL)	0.16 ± 0.37	0.15 ± 0.33	0.65	0.26	0.15
DCS (µg/dL)	0.41 ± 0.23	0.38 ± 0.24	0.74	0.48	− 0.01
AUC_g_ (µg/dLxh)	225 ± 104	218 ± 98	0.86	0.23	0.17

Continuous data are reported as means *±* standard deviation.

*BMI* body mass index, *HR* heart rate, *HRV* heart rate variability*,* *AW* awakening, *CAR* cortisol awakening response, *AUC*_g_ area under the curve with respect to ground, *DCS* diurnal cortisol slope.

**Fig. 1:**
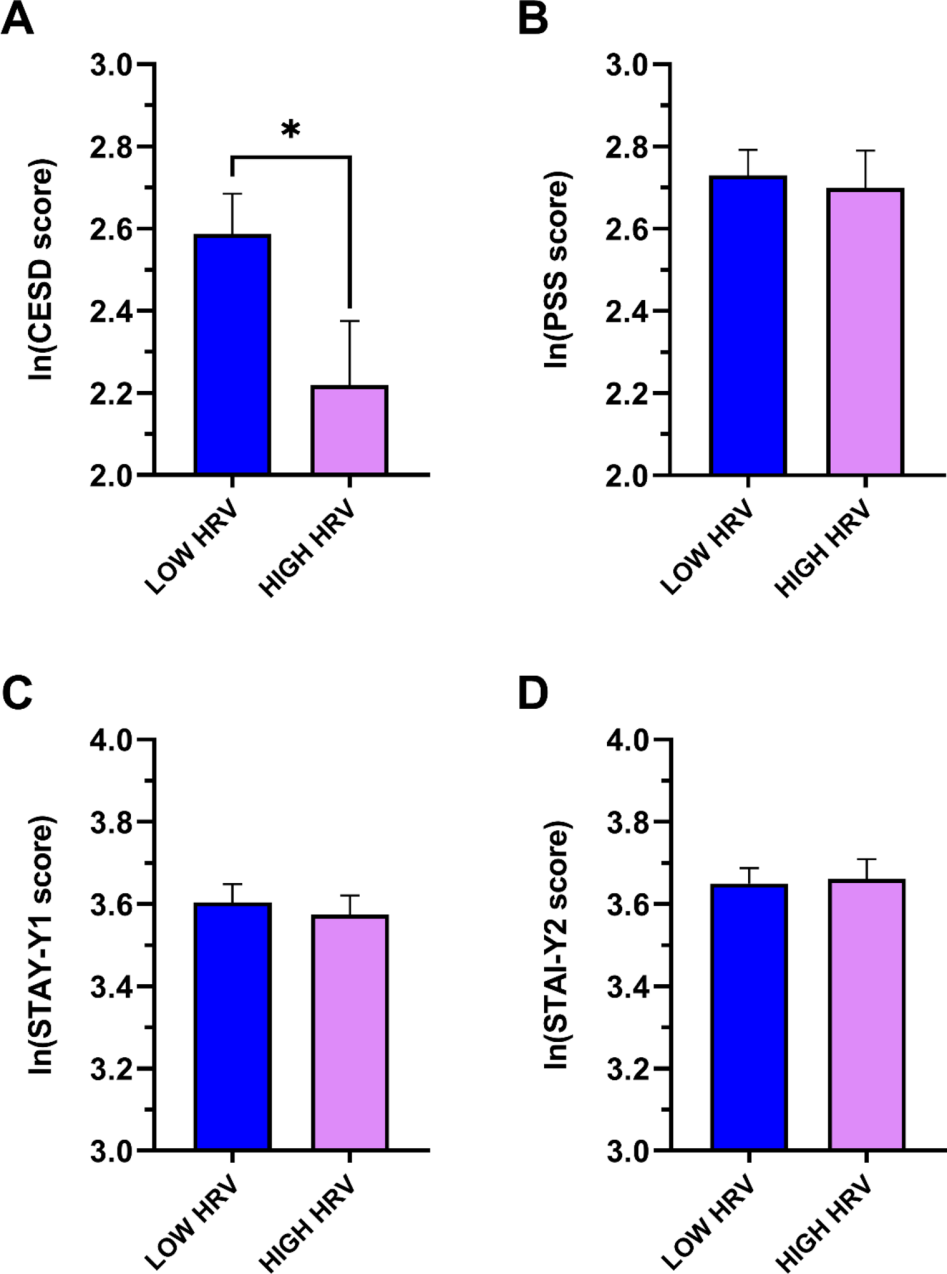
Psychometric characteristics of the LOW and HIGH HRV groups. Data are reported as mean ± standard error. * indicates a significant difference. *HRV* Heart Rate Variability, *Ln* natural logarithm, *CES-D* Center for Epidemiological Studies-Depression, *PSS* Perceived Stress Scale, *STAI* State-Trait Anxiety Inventory (Y1: State; Y2: Trait).

The psychometric characteristics of the two groups are shown in Fig. 1. The LOW HRV group showed a significantly greater depressive symptomatology (CES-D scale) compared with the HIGH HRV group (Fig. 1A), also when age was included as a covariate (F = 5.29, df = 1.72 *p* = 0.024). No significant differences were found between the two groups in other psychometric scores (Fig. 1B–D).

**Fig. 2:**
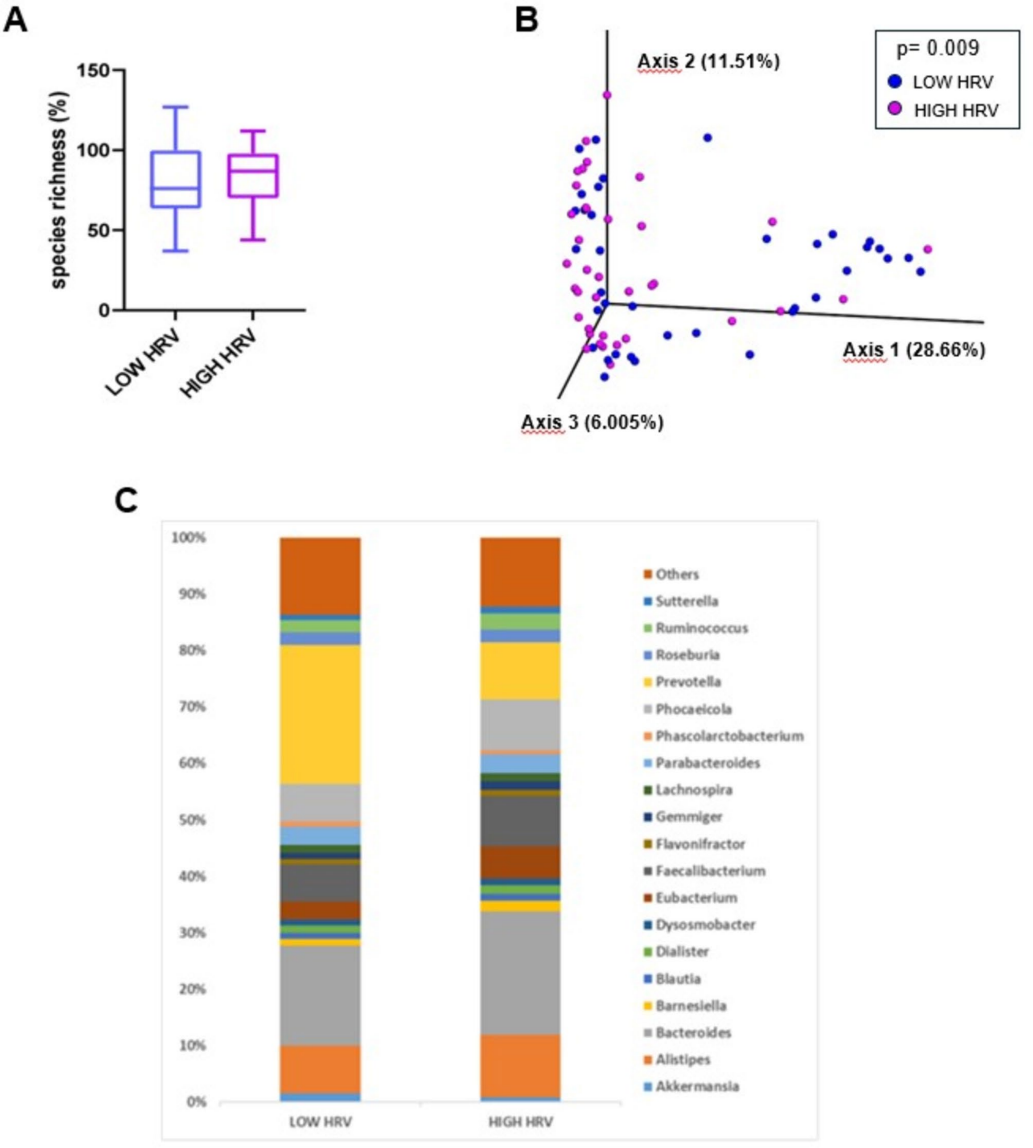
Evaluation of microbial diversity and composition in the LOW and HIGH HRV groups. **A** Whisker’s plot representing differences in species richness between the LOW and HIGH HRV group. The boxes are determined by the 25th and 75th percentiles. The whiskers are determined by 1.5 interquartile range (IQR). **B** Principal coordinate analyses of microbiota composition (beta diversity) conducted on fecal samples of LOW HRV and HIGH HRV subjects. **C** Taxonomic plots of all bacterial genera in the two groups. Only genera with a relative abundance > 1% are represented. *HRV* Heart Rate Variability.

Figure 2 represents the gut microbiota profile of the two groups. No significant differences were found between the LOW and HIGH HRV group in the percentage of microbial species richness (Fig. 2A). Then, a 3D PCoA was performed independently for each group to identify possible variations in the composition of gut microbiota (i.e., beta-diversity) (Fig. 2B). Interestingly, PERMANOVA analyses revealed a significant variation in microbiota composition between the LOW and the HIGH HRV group (*p* = 0.006). To characterize the microbiota composition of the two groups, we then represented bacterial genera with a relative abundance > 1% (Fig. 2C). Of these, six differed significantly between the two groups, with *Prevotella* being significantly more abundant in the LOW HRV group, and *Alistipes*, *Faecalibacterium*, *Eubacterium*, *Barnesiella* and *Gemmiger* being significantly less abundant in the LOW HRV group compared with the HIGH HRV group (Table 2). The abundance of other genera (*Fournierella* and *Mitsuokella*) differed significantly between the two groups, but their relative abundance in the entire sample was < 1%, (Table 2). To further investigate differences found at genus level, we found 6 species that were significantly more abundant and 7 species that were significantly less abundant in the LOW HRV compared with the HIGH HRV group (Table 3).

**Table 2:** Relative abundance (%) of bacterial genera that significantly differed between the LOW HRV (*n* = 37) and HIGH HRV (*n* = 38) group.

Genera	LOW HRV (%)	HIGH HRV (%)	*p*
Relative Abundance > 1%			
*Prevotella*	24.64 ± 4.76	10.15 ± 3.26	0.008
*Alistipes*	8.45 ± 1.17	10.95 ± 1.05	0.039
*Faecalibacterium*	6.51 ± 0.87	8.90 ± 0.90	0.017
*Eubacterium*	3.24 ± 0.5	5.73 ± 0.83	0.021
*Barnesiella*	1.16 ± 0.20	1.83 ± 0.24	0.019
*Gemmiger*	0.99 ± 0.17	1.54 ± 0.19	0.015
Relative Abundance < 1%			
*Fournierella*	0.05 ± 0.02	0.11 ± 0.02	0.016
*Mitsuokella*	0.18 ± 0.06	0.03 ± 0.02	0.022
*Culturomica*	0.04 ± 0.01	0.08 ± 0.01	0.017

Data related are reported as means ± SEM and were analyzed with the Mann-Whitney *U* test.

*HRV* Heart Rate Variability.

**Table 3:** Relative abundance (%) of bacterial species that significantly differed between the LOW HRV (*n* = 37) and HIGH HRV (*n* = 38) group.

Species	LOW HRV (%)	HIGH HRV (%)	*p*
*Alistipes communis*	0.28 ± 0.07	0.63 ± 0.09	0.001
*Alistipes onderdonkii*	1.06 ± 0.33	1.46 ± 0.31	0.003
*Culturomica unknown_species*	0.04 ± 0.01	0.08 ± 0.01	0.017
*Faecalibacterium prausnitzii*	2.87 ± 0.45	3.64 ± 0.42	0.029
*Faecalibacterium unknown_species*	3.64 ± 0.45	5.25 ± 0.52	0.013
*Fournierella unknown_species*	0.05 ± 0.02	0.11 ± 0.02	0.016
*Gemmiger formicilis*	0.41 ± 0.1	0.68 ± 0.01	0.008
*Prevotella copri*	17.02 ± 3.72	5.31 ± 2.31	0.009
*Prevotella lascolaii*	0.35 ± 0.14	0.09 ± 0.05	0.029
*Prevotellamassilia timonensis*	0.06 ± 0.02	0.01 ± 0.01	0.035
*Prevotella pectinovora*	0.03 ± 0.01	0.01 ± 0.01	0.031
*Prevotella unknown_species*	6.81 ± 1.44	3.36 ± 1.15	0.008

Data are reported as means ± SEM and were analyzed with the Mann-Whitney *U* test.

*HRV* Heart Rate Variability.

### Cortisol awakening response, psychometric characteristics, and microbiota profile

Using the median value of the CAR, participants were divided into LOW and HIGH CAR groups. There were no differences in terms of general characteristics and HRV between the two groups (see Supplementary Table S1 online). A flatter CAR in the LOW CAR group was mainly determined by significantly lower values of cortisol 30 min upon awakening (see Supplementary Table S1 online). The LOW CAR group also had a significantly greater DCS and significantly smaller AUC_g_ compared with the HIGH CAR group (see Supplementary Table S1 online).

**Fig. 3:**
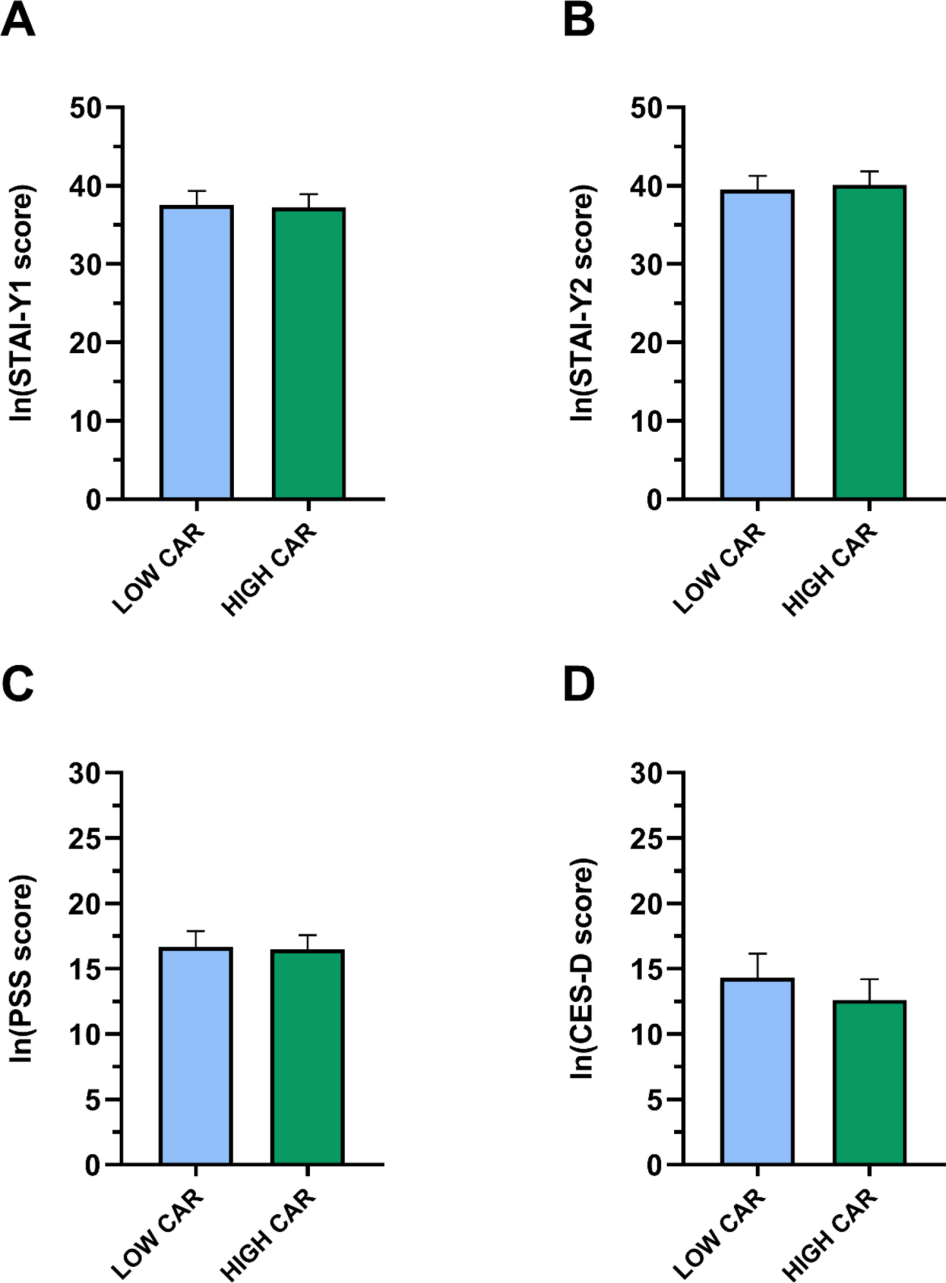
Psychometric characteristics of the LOW and HIGH CAR group. Data are reported as mean ± standard error. *CAR* Cortisol Awakening Response, *Ln* natural logarithm, *STAI* State-Trait Anxiety Inventory (Y1: State; Y2: Trait), *PSS* Perceived Stress Scale, *CES-D* Center for Epidemiological Studies-Depression.

As depicted in Fig. 3, no significant group differences were found in psychometric scores.

**Fig. 4:**
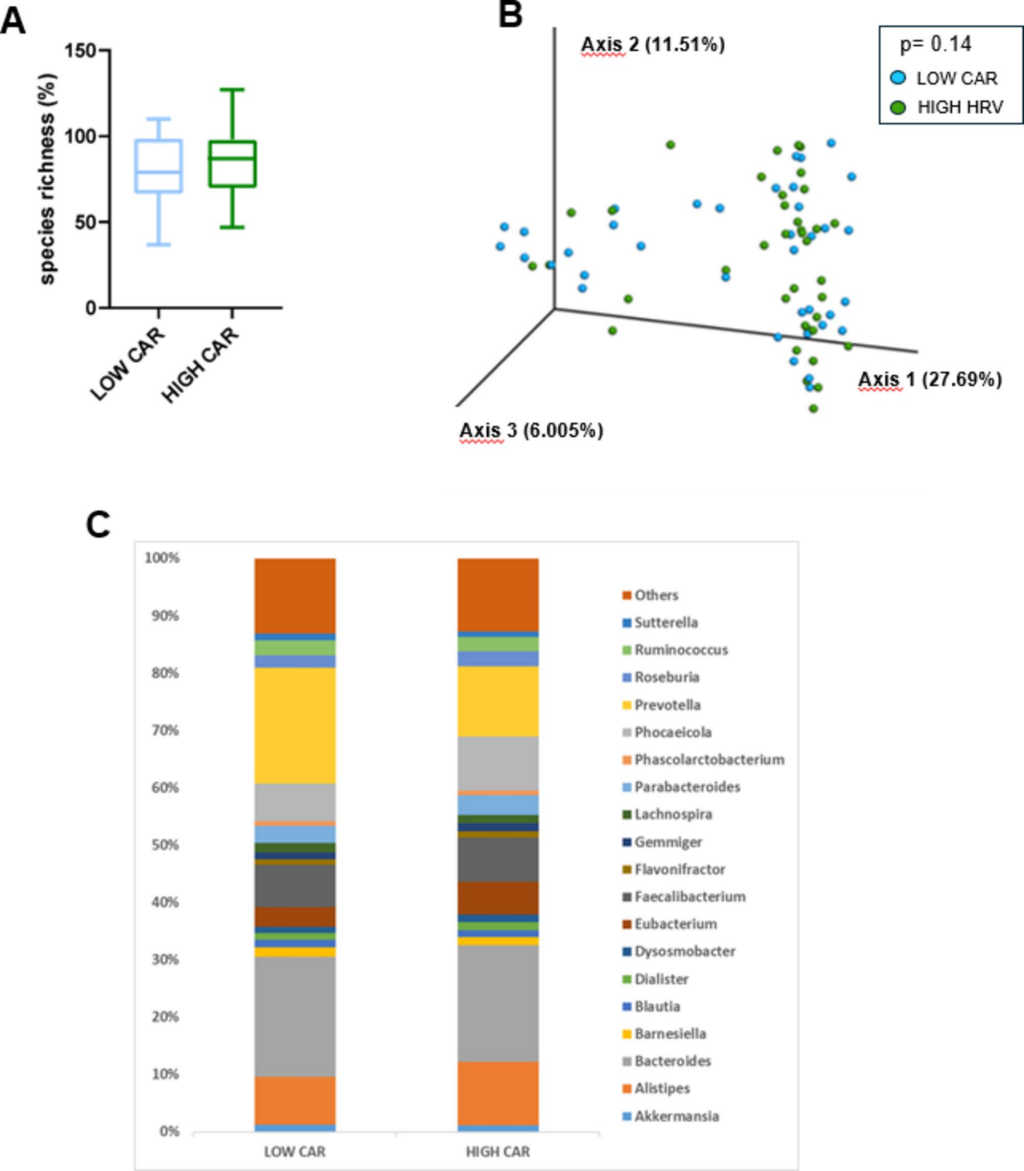
Evaluation of microbial diversity and composition in the LOW and HIGH CAR groups. **A** Whisker’s plot representing differences in species richness between the LOW and HIGH CAR group. The boxes are determined by the 25th and 75th percentiles. The whiskers are determined by 1.5 interquartile range (IQR). **B** Principal coordinate analyses of microbiota composition (beta diversity) conducted on fecal samples of LOW CAR and HIGH CAR subjects. **C** Taxonomic plots of all bacterial genera in the two groups. Only genera with a relative abundance > 1% are represented. *CAR* cortisol awakening response.

The percentage of species richness, as depicted in Fig. 4A, did not differ between the LOW and HIGH CAR group. Then, a 3D PCoA (Fig. 4B) was performed independently for each group to identify possible variations in the composition of gut microbiota (i.e., beta-diversity). PERMANOVA analyses did not reveal a significant variation in gut microbiota composition between the LOW and the HIGH CAR group (*p* = 0.137). There were no significant differences in the taxonomic composition and relative abundance of microbial groups at genus level, as depicted in Fig. 4C.

### Diurnal cortisol slope, psychometric characteristics, and microbiota profile

Using the median value of the DCS, participants were divided into LOW and HIGH DCS groups. There were no differences in terms of general characteristics and HRV between the two groups (see Supplementary Table S2 online). A smaller DCS was mainly determined by significantly lower values of cortisol upon awakening in the LOW DCS group (see Supplementary Table S2 online). The LOW DCS group also had significantly lower values of cortisol 30 min after awakening and a smaller AUC_g_ compared to the HIGH DCS group (see Supplementary Table S2 online).

**Fig. 5:**
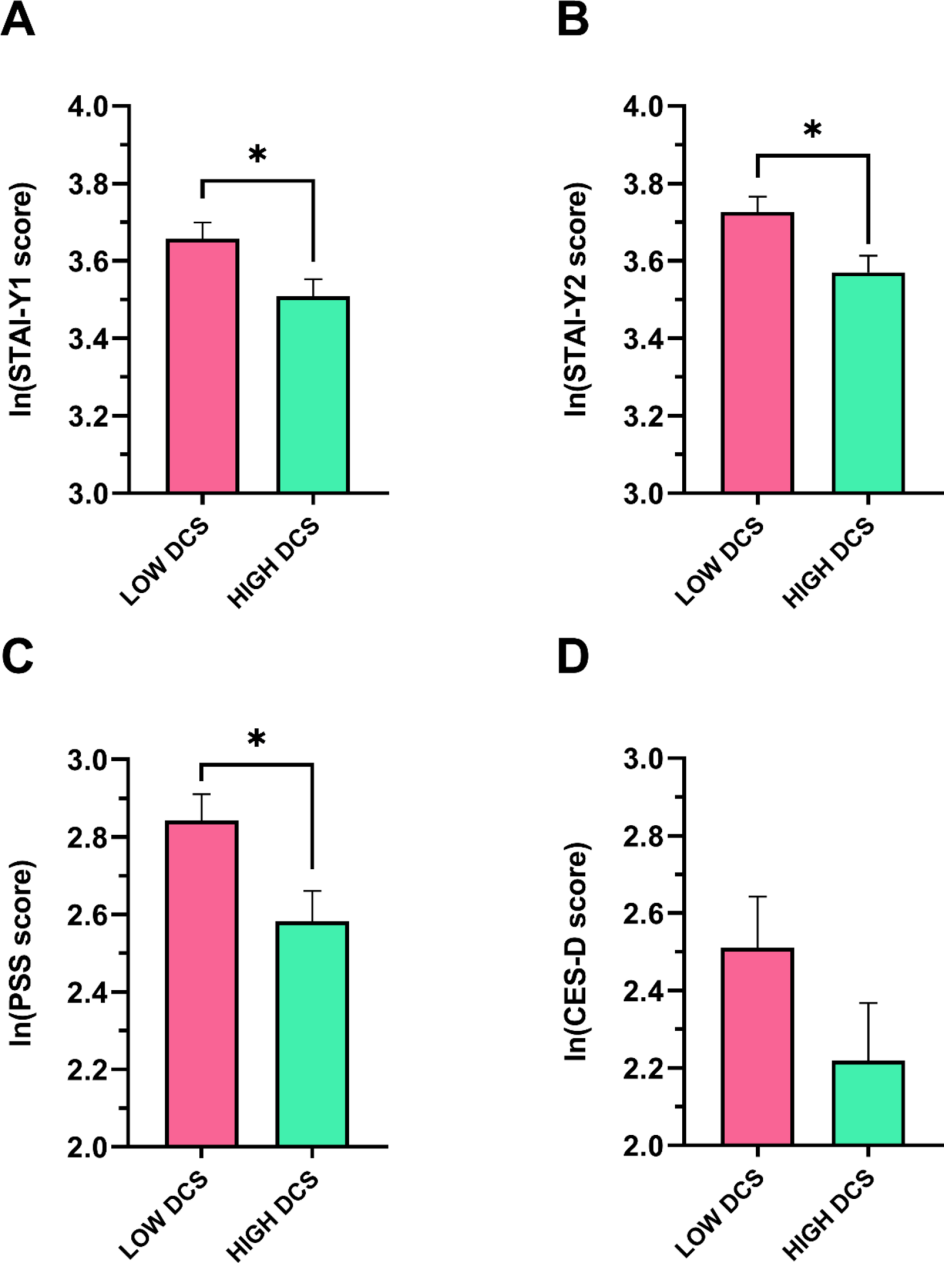
Psychometric characteristics of the LOW DCS and HIGH DCS groups. Data are reported as mean ± standard error. * Indicates a significant difference. *DCS* Diurnal Cortisol Slope, *Ln* natural logarithm, *STAI* State-Trait Anxiety Inventory (Y1: State; Y2: Trait), *PSS* Perceived Stress Scale, *CES-D* Center for Epidemiological Studies-Depression.

As depicted in Fig. 5, the LOW DCS group reported significant higher levels of state anxiety (STAI-Y1: t = 2.43, *p* < 0.05, Cohen’s d = 0.56), trait anxiety (STAI-Y2: t = 2.709, *p* < 0.05, Cohen’s d = 0.630), and perceived subjective stress (PSS, t = 2.504, *p* < 0.05, Cohen’s d = 0.578), while no significant difference was detected in depressive symptomatology (CES-D scale) between the two groups (Fig. 5).

**Fig. 6:**
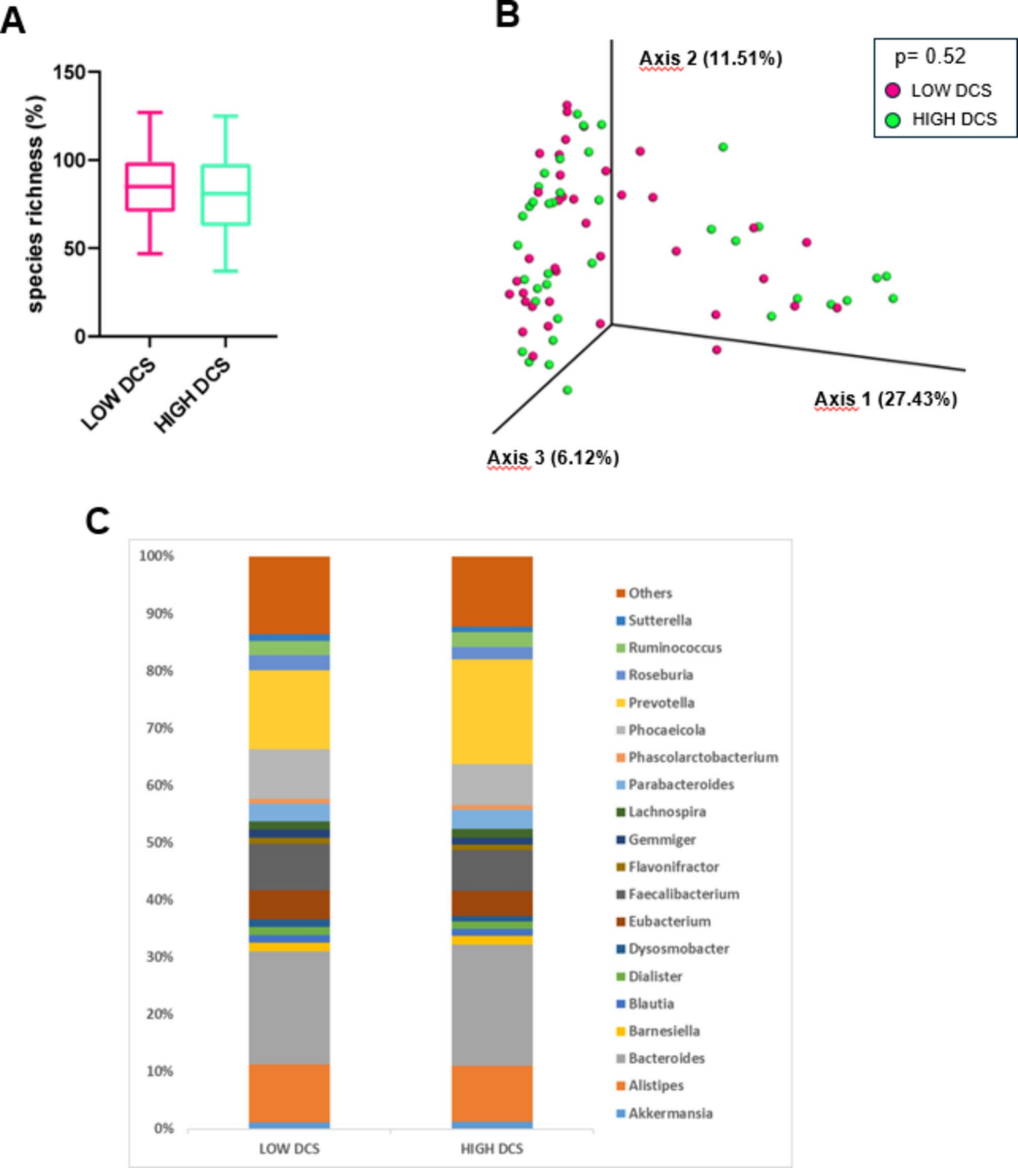
Evaluation of microbial diversity and composition in the LOW and HIGH DCS groups. **A** Whisker’s plot representing the difference in species richness between LOW and HIGH DCS group. The boxes are determined by the 25th and 75th percentiles. The whiskers are determined by 1.5 interquartile range (IQR). **B** Principal coordinate analyses of microbiota composition (beta diversity) conducted on fecal samples of LOW DCS and HIGH DCS subjects. **C** Taxonomic plots of all bacterial genera in the two groups. Only genera with a relative abundance > 1% are represented. *DCS* Diurnal Cortisol Slope.

The percentage of species richness, as depicted in Fig. 6A, did not differ between the LOW and HIGH DCS group. Then, a 3D PCoA (Fig. 6B) was performed independently for each group to identify possible variations in the composition of gut microbiota (i.e., beta-diversity). PERMANOVA analyses did not reveal a significant variation in microbiota composition between the LOW and HIGH DCS group (*p* = 0.518).

The taxonomic composition and relative abundance of microbial groups at genus level in the two groups is depicted in Fig. 6C. Among the genera with a relative abundance in the entire sample > 1%, only *Sutterella* was more abundant in the LOW compared with the HIGH DCS group, although this difference did not reach full statistical significance (Table 4). Other marginal differences between the two groups involved genera with a relative abundance of less than 1% in the entire sample (Table 4).

**Table 4:** Relative abundance (%) of bacterial genera that significantly differed between the LOW DCS (*n* = 37) and HIGH DCS (*n* = 38) group.

Genera	LOW DCS (%)	HIGH DCS (%)	*p*
Relative abundance > 1%			
*Sutterella*	1.23 ± 0.25	0.94 ± 0.32	0.078
Relative abundance < 1%			
*Parasutterella*	0.41 ± 0.13	0.67 ± 0.20	0.075
*Streptococcus*	0.14 ± 0.06	0.01 ± 0.01	0.066
*Mesosutterella*	0.03 ± 0.02	0.08 ± 0.04	0.089
*Holdemanella*	0.08 ± 0.04	Absent	0.063

Data are reported as means ± sem and were analyzed with the Mann-Whitney *U* test.

*DCS* diurnal cortisol slope.

### Overall daily cortisol, psychometric test and microbiota profile

Using the median value of the AUC_g_ as a measure of the overall production of cortisol throughout the day, participants were divided into LOW and HIGH AUC_g_ groups. There were no differences in general characteristics and HRV between the two groups (see Supplementary Table 3 S online). The LOW AUCg group had significantly lower values of cortisol at every assessment point (see Supplementary Table 3 S online).

**Fig. 7:**
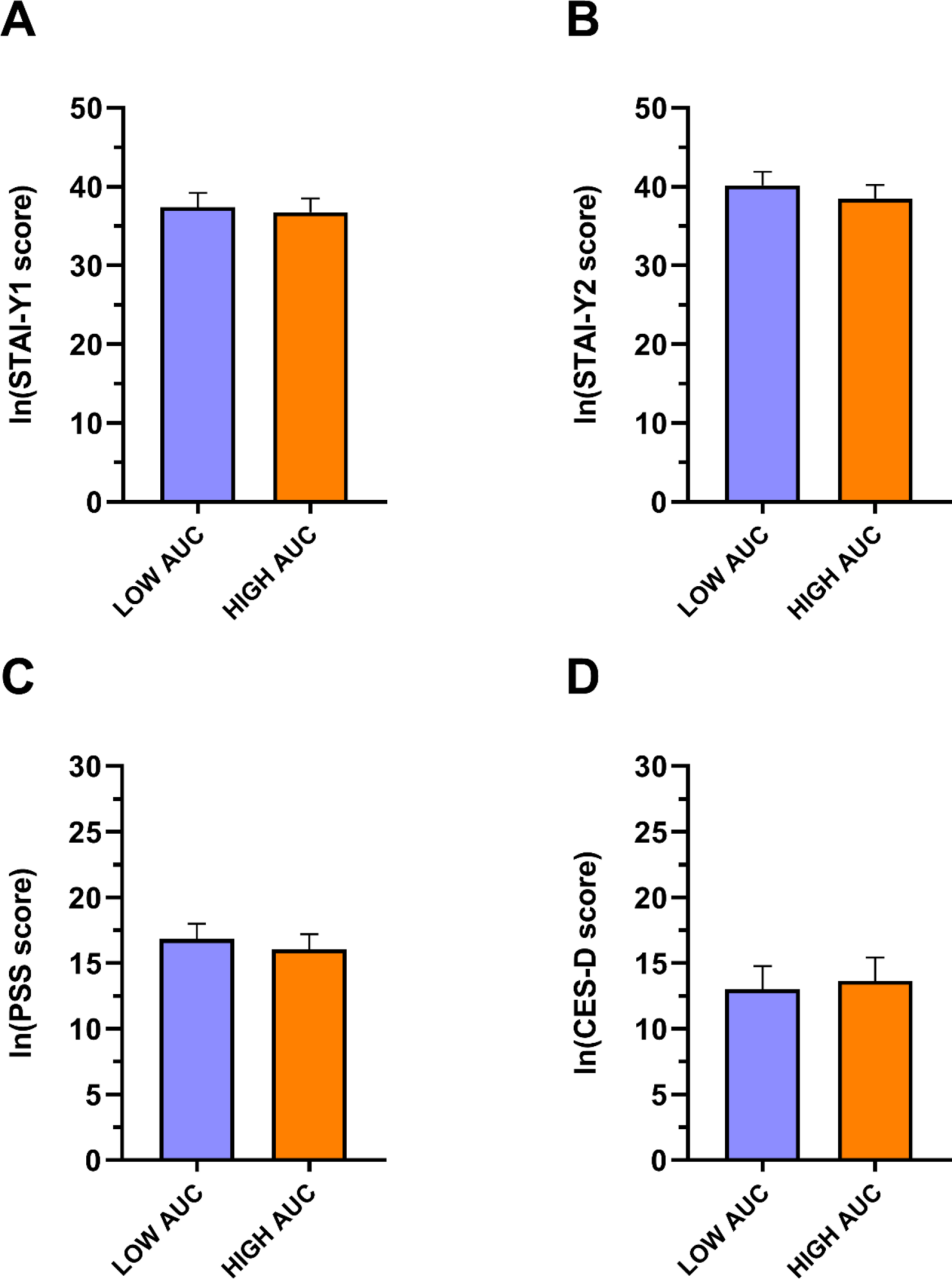
Psychometric characteristics of the LOW and HIGH AUC_g_ group. Data are reported as mean ± standard error. *AUC*_g_ area under the curve with respect to ground, *Ln* natural logarithm, *STAI* State-Trait Anxiety Inventory (Y1: State; Y2: Trait), *PSS* Perceived Stress Scale, *CES-D* Center for Epidemiological Studies-Depression.

As depicted in Fig. 7, no significant group differences were found in psychometric scores.

**Fig. 8:**
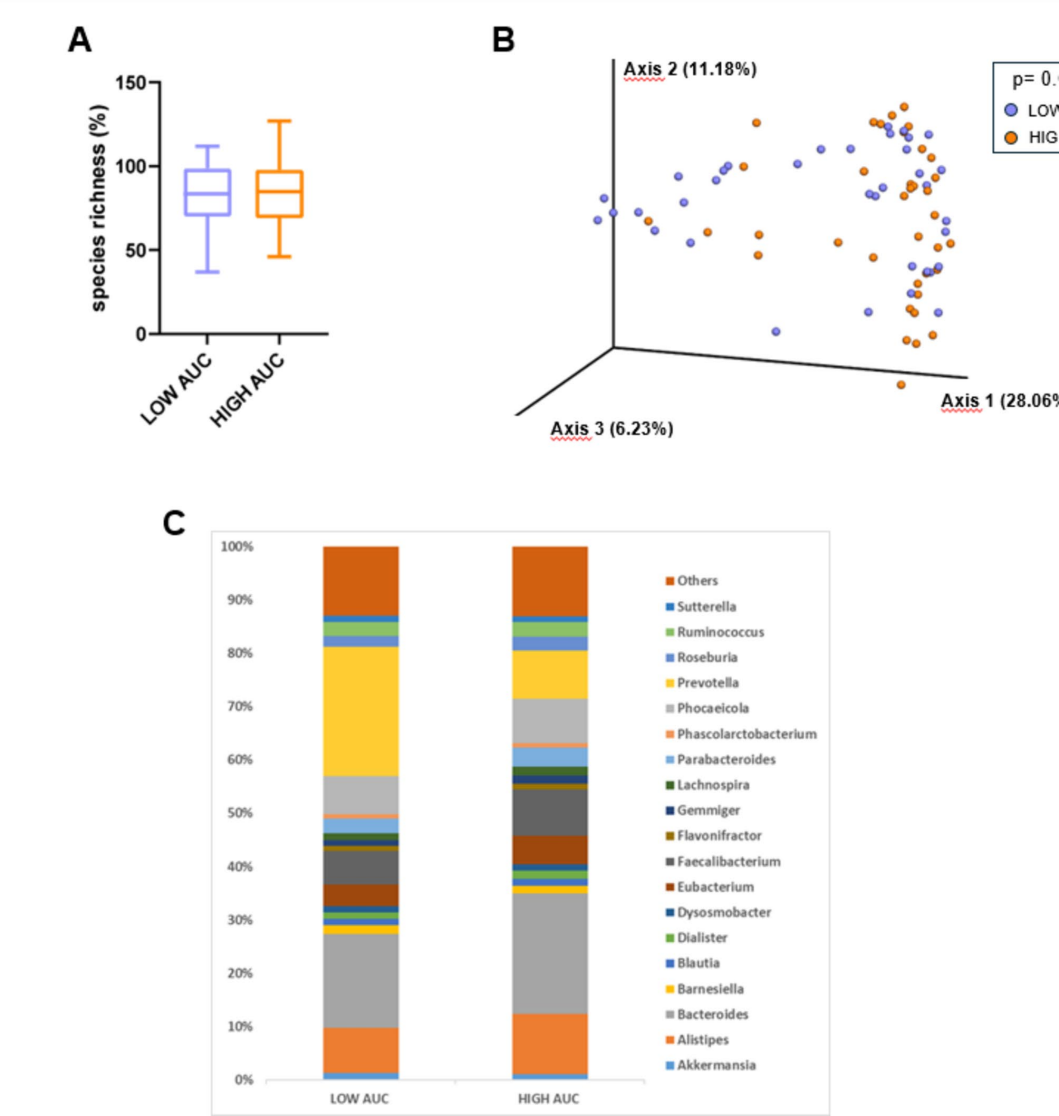
Evaluation of microbial diversity and composition in the LOW and HIGH AUC_g_ groups. **A** Whisker’s plot representing the difference in species richness between the LOW and HIGH AUC_g_ group. The boxes are determined by the 25th and 75th percentiles. The whiskers are determined by 1.5 interquartile range (IQR). **B** Principal coordinate analyses of microbiota composition (beta diversity) conducted on fecal samples of the LOW AUC_g_ and HIGH AUC_g_ group. **C** Bacterial genera that differ significantly between the LOW AUC_g_ and HIGH AUC_g_ group. *AUC*_g_ area under the curve with respect to ground.

The percentage of species richness, as depicted in Fig. 8A, did not differ between LOW and HIGH AUC_g_ groups. Then, a 3D PCoA (Fig. 8B) was performed independently for each group to identify possible variations in the composition of gut microbiota (i.e., beta-diversity). PERMANOVA analyses revealed a significant variation in microbiota composition between the LOW and the HIGH AUC_g_ group (*p* = 0.026). To characterize the gut microbiota composition of the two groups, we represented bacterial genera with a relative abundance > 1% (Fig. 8C). Among these, *Faecalibacterium* and *Blautia* were significantly less abundant in the LOW compared with the HIGH AUC_g_ group (Table 5). Other significant differences between the two groups involved microbial taxa (*Enterocloster*,* Lachnoclostridium*,* Escherichia*,* Parasutterella* and *Coprobacter*) with a relative abundance < 1% (Table 5). To further investigate differences found at genus level, we found one species that was significantly more abundant and 5 species that were significantly less abundant in the LOW AUC_g_ group (Table 6).

**Table 5:** Relative abundance (%) of bacterial genera that significantly differed between the LOW AUC_g_ (*n* = 37) and HIGH (*n* = 38) AUC_g_ group.

Genera	LOW AUC_g_(%)	HIGH AUC_g_ (%)	*p*
Relative abundance > 1%			
*Faecalibacterium*	6.20 ± 0.67	8.67 ± 0.95	0.045
*Blautia*	1.15 ± 0.13	1.38 ± 0.11	0.017
Relative abundance < 1%			
*Enterocloster*	0.22 ± 0.05	0.01 ± 0.04	0.015
*Lachnoclostridium*	0.05 ± 0.01	0.12 ± 0.02	0.001
*Escherichia*	0.41 ± 0.20	0.34 ± 0.20	0.042
*Parasutterella*	0.3 ± 0.09	0.69 ± 0.03	0.007
*Coprobacter*	0.07 ± 0.03	Absent	0.028

Data are reported as means ± SEM and were analyzed with the Mann-Whitney *U* test.

*AUC*_g_ area under the curve with respect to ground.

**Table 6:** Relative abundance (%) of bacterial species that significantly differed between the LOW AUC_g_ (*n* = 37) and HIGH AUC_g_ (*n* = 38) groups.

Species	LOW AUC_g_(%)	HIGH AUC_g_(%)	*p*
*Blautia wexlerae*	0.17 ± 0.05	0.30 ± 0.06	0.005
*Enterocloster unknown_species*	0.16 ± 0.02	0.24 ± 0.02	0.008
*Escherichia coli*	0.39 ± 0.19	0.33 ± 0.20	0.039
*Faecalibacterium unknown_species*	3.44 ± 0.37	5.07 ± 0.54	0.021
*Lachnoclostridium unknown_species*	0.05 ± 0.01	0.12 ± 0.02	0.001
*Parasutterella excrementihominis*	0.17 ± 0.05	0.50 ± 0.18	0.002

Data are reported as means ± SEM and were analyzed with the Mann-Whitney *U* test.

*AUC*_g_ area under the curve with respect to ground.

## Discussion

To the best of our knowledge, this is the first study investigating the extent to which resting measures of vagally-mediated HRV and salivary cortisol parameters are associated with specific psychometric characteristics and gut microbiota composition in a sample of healthy adults. We used a categorical approach based on the median split of HRV and salivary cortisol parameters to characterize groups in terms of psychometric characteristics and gut microbiota profile.

### Heart rate variability, psychometric characteristics, and gut microbiota profile

Subjects with low values of resting vagally-mediated HRV showed higher scores in depressive symptomatology compared with subjects with high vagally-mediated HRV, in agreement with studies showing that depression is associated with low indexes of cardiac vagal modulation^35,36^. No differences were found in other psychometric scores, suggesting that low HRV may specifically represent a biomarker of vulnerability to depressive symptoms in healthy adults, as previously reported^37^.

No differences were found between the LOW and HIGH HRV groups in overall gut microbiota richness (alpha diversity), whereas other studies found a positive correlation between alpha diversity and cardiac vagal activity^10,11^. Greater bacterial diversity is frequently linked to better health outcomes, even though the utility of alpha diversity as a universal indicator of gut health remains a topic of debate^38^. In this study, PCoA highlighted differences in gut microbiota composition (beta-diversity) between the LOW and HIGH HRV group. Specifically, among the genera with a relative abundance > 1%, subjects with low HRV had a higher abundance of *Prevotella*, a genus commonly associated with chronic inflammatory diseases^39^, and more in detail a higher abundance of *Prevotella copri* at the species level. Notably, a greater abundance of *Prevotella*was also previously reported in the faecal samples of a small group of patients with depression^40^. On the other hand, we found that *Faecalibacterium*,* Alistipes*,* Eubacterium*,* Barnesiella*, and *Gemmiger* were less abundant in the LOW HRV group. This is in line with a previous study showing a smaller *Faecalibacterium*abundance in women with low HRV and higher depressive scores^11^. Additionally, other studies have reported reduced *Faecalibacterium*levels in patients with major depressive disorder^41,42^. In our study, we were able to identify at the species level that *Faecalibacterium prausnitzii* was less abundant in the LOW HRV group. *Faecalibacterium prausnitzii*is a key butyrate-producing bacterium associated with gut health and reduced inflammation^43^, and a reduction in its relative abundance has been linked to an increased risk of postoperative ileal Crohn’s disease recurrence^44^. Notably, *Faecalibacterium prausnitzii*is currently under consideration as a promising new-generation probiotic and auxiliary diagnostic biomarker of depression^45^. As for the *Alistipes*genus, there is contrasting evidence: some studies suggest that it may have protective effects against diseases like liver fibrosis and colitis, while others associate it with colorectal cancer and depressive symptoms^46,47^. It is also important to note that in the current study individuals with LOW HRV and greater depressive symptoms showed a lower abundance of *Gemmiger*, which is consistent with previous studies in patients with major depressive disorder compared to healthy controls^48^. The *Eubacterium*genus, which was less abundant in the LOW HRV group, plays a key role in inflammation modulation, immune regulation, and gut barrier maintenance^49^. Although inconsistent results on the association between *Eubacterium*abundance and HRV have been reported^10^, a smaller abundance of this bacterial genus was found in individuals with major depressive disorder^50^. Finally, studies on *Barnesiella*are limited and yield conflicting results. Some investigations suggest that reduced levels of this genus are linked to vascular disease^51^, while others report a larger predominance of *Barnesiella* in psychiatric patients compared to healthy controls. To summarize, in the current study individuals with low values of resting HRV showed greater depressive symptoms. Moreover, the composition of their gut microbiota - specifically a higher abundance of *Prevotella* and a smaller abundance of *Faecalibacterium*,* Alistipes*, and *Gemmiger*– resembles previous findings in patients with depression. Notably, earlier studies have indicated that for daytime HRV (RMSSD index), values below 25 ms are associated with elevated general health risk and depression risk^52,53^. In the current study the median split for RMSSD-HRV was 34 ms, but unfortunately the relatively small sample size did not allow us to consider the 25 ms cutoff with enough statistical power. Nevertheless, the current findings suggest that low HRV may play an important role in the bidirectional link between gut dysbiosis and depression.

### Salivary cortisol, psychometric characteristics, and gut microbiota profile

To date, no studies have examined daily measures of salivary cortisol in association with psychometric characteristics and gut microbiota composition in healthy adults. In this study, we considered three key parameters of diurnal cortisol variations: the cortisol awakening response (CAR), indicating the increase in cortisol levels upon awakening; the diurnal cortisol slope (DCS), reflecting the cortisol decrease from morning to evening; and the area under the curve with respect to ground AUC_g_), representing overall cortisol daily production^54^. These parameters have implications for human functioning and health, with a flatter DCS associated with poor physical and mental health^55^, and a smaller AUC_g_linked to conditions of allostatic load^56^. Furthermore, while a blunted CAR has been associated with post-traumatic stress disorder, a greater CAR was found to predict the onset of major depression and anxiety disorders^55,57^. Among these cortisol parameters, only the median split of DCS separated the sample in two groups showing significant differences in psychometric scores, whereby individuals with a flatter DCS (i.e., LOW DCS group) reported higher state and trait anxiety and higher perceived stress. Nevertheless, no significant differences in gut microbiota composition were found between the LOW and HIGH DCS groups. Among the genera with a relative abundance > 1%, we only observed a tendency for a greater abundance of *Suttarella*in the LOW DCS group. Interestingly, this genus has been previously associated with generalized anxiety disorder^58^, hinting at a possible implication of *Suttarella* in the link between flatter DCS and vulnerability to anxiety.

On the other hand, neither the median split of CAR nor AUC_g_ separated the sample in two groups with differences in psychometric scores. In terms of gut microbiota composition, we did not find any difference between the LOW CAR and HIGH CAR group. However, we found a significant difference in gut microbiota composition (beta-diversity) between the LOW AUC_g_ and HIGH AUC_g_ group. Specifically, among the genera with a relative abundance > 1%, individuals in the LOW AUC_g_ showed a lower relative abundance of *Faecalibacterium*, similarly to what was observed for individuals in the LOW HRV group (see above).

### Conclusion

The current study investigated the extent to which vagally mediated HRV and salivary cortisol parameters were associated with specific psychological characteristics and gut microbiota features in an Italian community sample of healthy adults. Using a categorical approach based on the median split of these physiological parameters, we identified an interesting association between low vagally-mediated HRV, greater depressive symptomatology, and altered gut microbiota. The vagus nerve, linking brain and gut through its afferent and efferent branches, is a critical route in the bidirectional communication of the microbiota-gut brain axis^59^. Directly or indirectly, vagus afferent fibers can sense and relay gut microbiota signals to the brain, including the central autonomic network^8^. This may result in alterations in autonomic nervous system function and reduced HRV, which characterize mental health disorders such as depression^35^. On the other hand, low vagal efferent activity (indexed by reduced HRV) may result in gut hyperpermeability and inflammation, which could influence relative bacterial abundance and negatively impact gut health^8^. Therefore, our HRV results support the involvement of the vagus nerve in the microbiota-gut-brain axis. On the other hand, different cortisol parameters were associated either with higher anxiety and perceived stress (DCS), or with differences in gut microbiota profile (AUC_g_). It may be that the association of cortisol indices with psychometric characteristics and gut microbiota composition is weaker, and our median split approach did not have enough power to capture them statistically. Hence, future studies should consider other statistical approaches (i.e., Bayesian analyses) to further investigate the utility of daily cortisol indices as biomarkers of microbiota-gut brain axis function.

In interpreting the current results, some limitations must be acknowledged. First, this research represents an initial hypothesis testing and should be used to plan larger confirmatory studies. Second, as the present study was cross-sectional, it is not possible to interpret the results in terms of cause-effect relations. Future longitudinal studies are required to determine whether low vagally-mediated HRV at rest may constitute an early biomarker of vulnerability to depression and gut microbiota alterations. Also, in this study we did not control for individual dietary habits, and we did not collect any information on menstrual cycle phase from the female sample, which could have influenced cortisol parameters as demonstrated by recent meta-analytic findings showing that women in the follicular phase have higher cortisol levels than women in the luteal phase^60^. Relatedly, future studies should consider the possibility to collect saliva samples over more than only one day, since at least 4–10 days are recommended to reliably measure parameters of diurnal cortisol rhythm^61^. Lastly, participants were recruited within a small urban area, therefore generalizability should be tested in diverse populations and settings. Despite these limitations, the current results provide new insight into the utility of vagally-mediated HRV as a proxy measure of vagal efferent activity for the study of microbiota-gut brain axis disturbances.

## Electronic supplementary material

Below is the link to the electronic supplementary material.

Supplementary Material 1.

## Data Availability

The datasets used during the current study will be made available from the corresponding author on reasonable request.
