# Supplementary material for: Heart rate variability, daily cortisol indices and their association with psychometric characteristics and gut microbiota composition in an Italian community sample
Source: Sci Rep. 2025 Mar 12;15:8584. doi: 10.1038/s41598-025-93137-8 (PMC11903775; doi:10.1038/s41598-025-93137-8)
Supplement: Supplementary file 1 — Supplementary Material 1. [file 41598_2025_93137_MOESM1_ESM.docx]

**Heart rate variability, daily cortisol indices and their association with psychometric characteristics and gut microbiota composition in an Italian community sample**

**Supplementary Table S1.** Participant characteristics for the LOW CAR (n = 37) and HIGH CAR (n = 38) group.

|  | LOW CAR | HIGH CAR | t/χ^2^ | p | d |
| --- | --- | --- | --- | --- | --- |
| Self-reported gender | F = 19 M =18 | F = 19 M = 19 | 0.01 | 0.9 |  |
| Age (years) | 38.8 ± 14.8 | 36.2 ± 13.8 | 0.8 | 0.21 | 0.19 |
| Smokers (n) | 11 | 11 | 0 | 1 |  |
| BMI (Kg*m^-2^) | 23.8 ± 4.2 | 23.4 ± 3.9 | 0.38 | 0.35 | 0.08 |
| HR (bpm) | 70.7 ± 8.6 | 69.1 ± 11.4 | 0.84 | 0.2 | 0.19 |
| HRV (ms) | 33.3 ± 16.9 | 37.6 ± 19.5 | -1.0 | 0.15 | -0.24 |
| Cortisol AW (μg/dL) | 0.54 ± 0.24 | 0.46 ± 0.25 | 1.26 | 0.1 | 0.292 |
| Cortisol AW+30min (μg/dL) | 0.43 ± 0.20 | 0.91 ± 0.38 | -6.74 | <0.001 | -1.56 |
| Cortisol 12:00 (μg/dL) | 0.22 ± 0.14 | 0.26 ± 0.17 | -1.25 | 0.108 | -0.75 |
| Cortisol 22:00 (μg/dL) | 0.10 ± 0.07 | 0.11 ± 0.05 | -0.39 | 0.345 | -0.09 |
| CAR (μg/dL) | -0.11 ± 0.13 | 0.45 ± 0.33 | -9.56 | <0.001 | -2.19 |
| DCS (μg/dL) | 0.44 ± 0.22 | 0.35 ± 0.23 | -7.1 | <0.001 | -1.63 |
| AUC_g_ (μg/dLxh) | 182 ± 83 | 264 ± 98 | -3.55 | <0.001 | -0.826 |

Notes. Continuous data are reported as means *±* standard deviation. Abbreviations: CAR: Cortisol Awakening Response; BMI: body mass index; HR: Heart Rate; HRV: Heart Rate Variability; AW: Awakening; DCS: Diurnal Cortisol Slope; AUC_g_: Area Under the Curve with respect to ground.

**Supplementary Table S2.** Participant characteristics for the LOW DCS (n = 37) and HIGH DCS (n = 38) group.

|  | LOW DCS | HIGH DCS | t/χ^2^ | p | d |
| --- | --- | --- | --- | --- | --- |
| Self-reported gender | F = 20 M = 17 | F = 18 M =2 0 | 0.47 | 0.491 |  |
| Age (years) | 37.4 ± 14.4 | 37.2 ± 14.3 | 0.04 | 0.48 | 0.02 |
| Smokers (n) | 13 | 9 | 1.03 | 0.30 |  |
| BMI (Kg*m^-2^) | 23.6 ± 4.2 | 23.7± 3.8 | -0.14 | 0.45 | -0.03 |
| HR (bpm) | 68.2 ± 9.4 | 71.1 ± 10.5 | -1.27 | 0.1 | -0.29 |
| HRV (ms) | 39.3 ± 18.2 | 33.1 ± 19.0 | 1.4 | 0.08 | 0.33 |
| Cortisol AW (μg/dL) | 0.32 ± 0.09 | 0.68 ± 0.22 | -9.16 | <0.001 | -2.1 |
| Cortisol AW+30min (μg/dL) | 0.55 ± 0.32 | 0.79 ± 0.41 | -2.71 | 0.004 | -0.63 |
| Cortisol 12:00 (μg/dL) | 0.22 ± 0.12 | 0.26 ± 0.18 | -0.98 | 0.16 | -0.23 |
| Cortisol 22:00 (μg/dL) | 0.10 ± 0.07 | 0.11 ± 0.06 | -0.51 | 0.3 | -0.12 |
| CAR (μg/dL) | 0.23 ± 0.33 | 0.11 ± 0.40 | 1.28 | 0.102 | 0.29 |
| DCS (μg/dL) | 0.21 ± 0.09 | 0.56 ± 0.19 | -2.84 | 0.003 | -0.65 |
| AUC_g_ (μg/dLxh) | 195 ± 75 | 247 ± 111 | -2.48 | 0.008 | -0.57 |

Notes. Continuous data are reported as means *±* standard deviation. Abbreviations: DCS: Diurnal Cortisol Slope; BMI: body mass index; HR: Heart Rate; HRV: Heart Rate Variability; AW: Awakening; CAR: Cortisol Awakening Response; AUC_g_: Area Under the Curve with respect to ground.

**Supplementary Table S3.** Participant characteristics for the LOW AUC_g_ (n = 37) and HIGH AUC_g_ (n = 38) group.

|  | LOW AUC_g_ | HIGH AUC_g_ | t/χ^2^ | p | d |
| --- | --- | --- | --- | --- | --- |
| Self-reported gender | F = 20 M = 17 | F = 18 M = 20 | 0.33 | 0.56 |  |
| Age (years) | 40 ± 15.4 | 35.8 ± 13.0 | 1.25 | 0.11 | 0.29 |
| Smokers (n) | 12 | 10 | 0.33 | 0.74 |  |
| BMI (Kg*m^-2^) (SD) | 23.9 ± 3.8 | 23.3 ± 4.3 | 0.59 | 0.28 | 0.14 |
| HR (bpm) | 67.9 ± 9.4 | 71.3 ± 10.6 | -1.49 | 0.07 | -0.35 |
| HRV (ms) | 36.6 ± 17.8 | 33.8 ± 18.7 | 0.78 | 0.22 | 0.19 |
| Cortisol AW (μg/dL) | 0.45 ± 0.20 | 0.55 ± 0.27 | -1.99 | 0.05 | -0.47 |
| Cortisol AW+30min (μg/dL) | 0.44 ± 0.21 | 0.90 ± 0.39 | -6.1 | <0.001 | -1.4 |
| Cortisol 12:00 (μg/dL) | 0.16 ± 0.07 | 0.32 ± 0.18 | -5.29 | <0.001 | -1.22 |
| Cortisol 22:00 (μg/dL) | 0.07 ± 0.04 | 0.14 ± 0.06 | -5.45 | <0.001 | -1.27 |
| CAR (μg/dL) | -0.01 ± 0.20 | 0.34 ± 0.42 | -4.38 | <0.001 | -1.01 |
| DCS (μg/dL) | 0.38 ± 0.20 | 0.41 ± 0.26 | -5.05 | <0.001 | -1.17 |
| AUC_g_ (μg/dLxh) | 148 ± 45 | 295 ± 81 | -9.65 | <0.001 | 2.24 |

Notes. Continuous data are reported as means *±* standard deviation. Abbreviations: AUC_g_: Area Under the Curve with respect to ground; BMI: body mass index; HR: Heart Rate; HRV: Heart Rate Variability; AW: Awakening; CAR: Cortisol Awakening Response; DCS: Diurnal Cortisol Slope.
